# A Novel Chloroplast Protein RNA Processing 8 Is Required for the Expression of Chloroplast Genes and Chloroplast Development in *Arabidopsis thaliana*

**DOI:** 10.3389/fpls.2021.700975

**Published:** 2021-12-09

**Authors:** Mengmeng Kong, Yaozong Wu, Ziyuan Wang, Wantong Qu, Yixin Lan, Xin Chen, Yanyun Liu, Perveen Shahnaz, Zhongnan Yang, Qingbo Yu, Hualing Mi

**Affiliations:** ^1^National Key Laboratory of Plant Molecular Genetics, CAS Center for Excellence in Molecular Plant Sciences, Shanghai Institute of Plant Physiology and Ecology, Chinese Academy of Sciences, Shanghai, China; ^2^Shanghai Key Laboratory of Plant Molecular Sciences, College of Life Sciences, Shanghai Normal University, Shanghai, China

**Keywords:** *Arabidopsis*, chloroplast, plastid-encoded RNA polymerase, RpoA, RNA processing 8

## Abstract

Chloroplast development involves the coordinated expression of both plastids- and nuclear-encoded genes in higher plants. However, the underlying mechanism still remains largely unknown. In this study, we isolated and characterized an *Arabidopsis* mutant with an albino lethality phenotype named *RNA processing 8* (*rp8*). Genetic complementation analysis demonstrated that the gene *AT4G37920* (*RP8*) was responsible for the mutated phenotype. The *RP8* gene was strongly expressed in photosynthetic tissues at both transcription and translation protein levels. The RP8 protein is localized in the chloroplast and associated with the thylakoid. Disruption of the *RP8* gene led to a defect in the accumulation of the *rpoA* mature transcript, which reduced the level of the RpoA protein, and affected the transcription of PEP-dependent genes. The abundance of the chloroplast rRNA, including *23S*, *16S*, *4.5S*, and *5S rRNA*, were reduced in the *rp8* mutant, respectively, and the amounts of chloroplast ribosome proteins, such as, PRPS1(uS1c), PRPS5(uS5c), PRPL2 (uL2c), and PRPL4 (uL4c), were substantially decreased in the *rp8* mutant, which indicated that knockout of RP8 seriously affected chloroplast translational machinery. Accordingly, the accumulation of photosynthetic proteins was seriously reduced. Taken together, these results indicate that the RP8 protein plays an important regulatory role in the *rpoA* transcript processing, which is required for the expression of chloroplast genes and chloroplast development in *Arabidopsis*.

## Introduction

In higher plants, the chloroplast is a kind of semi-autonomous organelle that originated from a free-living cyanobacterium and has retained a reduced genome during the evolutionary process (Raven and Allen, 2003). Chloroplasts as the typical plastids in leaf mesophyll cells develop from protoplastids (Valkov et al., 2009). It is estimated that over 3,000 proteins exist in the chloroplast (Sato et al., 1999; Leister, 2003). However, most of the chloroplast proteins are encoded by the nuclear genes and imported from the cytosol (Inaba and Schnell, 2008). The chloroplast genome only encodes 120–130 genes that primarily participate in photosynthesis, plastid transcription, and translation processes (Daniell et al., 2016). Thus, chloroplast development involves the coordinated expression of both plastids- and nuclear-encoded genes (Leister, 2003; Lopez-Juez and Pyke, 2005).

Chloroplast genes are organized as operons which are transcribed as polycistronic units by two RNA polymerases, namely, a nuclear-encoded phage-type RNA polymerase (NEP) and a plastid-encoded bacterial-type RNA polymerase (PEP) (Pfalz and Pfannschmidt, 2013; Yu et al., 2014a; Pfannschmidt et al., 2015). NEP is involved in the transcription of housekeeping genes when plant cells establish the plastid transcription system during the early stage of chloroplast development. While, PEP is responsible for the transcription of photosynthetic-related genes (Pfalz and Pfannschmidt, 2013; Chi et al., 2015). In the chloroplast, the PEP complex represents a major RNA polymerase activity; over 80% of all primary chloroplast genes are transcribed by PEP in mature green leaves (Zhelyazkova et al., 2012). Although the core subunits of the PEP enzyme (α, β, β′, and β″) are encoded by plastid-encoded genes *rpoA*, *rpoB*, *rpoC1*, *rpoC2*, respectively, numerous nuclear-encoded accessory proteins have been identified from the PEP complex in recent years (Pfalz et al., 2006; Yu et al., 2014a), which play important regulatory roles in maintaining PEP transcriptional activity. For example, sigma factors that are encoded by the nuclear genome confer promoter recognition for PEP-dependent transcription (Schweer et al., 2006, 2010). In addition, 12 nucleus-encoded regulatory proteins called PEP-associated proteins (PAPs) have been identified by precise biochemical techniques, which are tightly associated with the PEP core subunits (Steiner et al., 2011; Pfalz and Pfannschmidt, 2013). They perform specific functions in the PEP complex, including protecting chloroplast nucleoids from superoxide anion radicals and redox reactions (Pfalz et al., 2006; Arsova et al., 2010; Pfalz and Pfannschmidt, 2013). pTAC10/PAP3 which co-migrates with RpoB can be phosphorylated by chloroplast-targeted casein kinase 2 (cpCK2) to regulate the transcription of plastid genes (Yu et al., 2018). TAC7/PAP12 interacts with FLN1, TAC10/PAP3, TAC12/PAP5, and TAC14/PAP7, regulating chloroplast gene expression (Yu et al., 2013). Interestingly, *paps* knockout mutants always exhibit an albino or pale-green phenotype with impaired transcription of PEP-dependent genes, suggesting that loss of PAPs protein have affected the activity of the PEP complex (Pfalz et al., 2006; Arsova et al., 2010; Gao et al., 2011; Steiner et al., 2011; Yu et al., 2013).

Chloroplast gene expression is regulated at various levels including transcription, RNA metabolism, and translation (Pfannschmidt and Liere, 2005; del Campo, 2009). After chloroplast transcript precursor is produced, it undergoes post-transcriptional processes including exo- and endo-ribonuclease cleavage, RNA splicing, and editing (Bollenbach et al., 2005). Numerous nuclear-encoded chloroplast ribonucleases (RNases) in *Arabidopsis* have been verified to be involved in RNA processing and degradation (MacIntosh and Castandet, 2020), such as exoribonucleases PNPase, RNase R, and RNR1 (Bollenbach et al., 2005; Germain et al., 2011, 2012), endoribonuclease RNase E (Mudd et al., 2008; Walter et al., 2010), CSP41a and CSP41b (Beligni and Mayfield, 2008; Chevalier et al., 2015). Also, the pentatricopeptide repeat (PPR) protein family has been found to be involved in different aspects of RNA metabolism in chloroplasts, including RNA transcription and stability, RNA editing, RNA maturation, RNA translation, and RNA splicing (Schmitz-Linneweber and Small, 2008; Zoschke et al., 2016; Wang et al., 2021). For example, PDM1 is involved in the processing of *rpoA* through association with the polycistronic mRNA in *Arabidopsis* (Wu and Zhang, 2010; Zhang et al., 2015). Additionally, the PDM1 protein is also involved in plastid RNA editing events (Pyo et al., 2013; Zhang et al., 2015). *Arabidopsis* YS1 is required for editing *rpoB* transcripts (Zhou et al., 2009). OTP70 is involved in splicing of the *rpoC1* transcript, which causes a typical PEP-deficient phenotype (Chateigner-Boutin et al., 2011). CLB19 is required for editing the *rpoA* transcript (Chateigner-Boutin et al., 2008). OsPPR16 is responsible for the editing of *rpoB* in rice, knockout of *OsPPR16* leads to impaired accumulation of the RpoB protein and reduced expression of PEP-dependent genes (Huang et al., 2020). These mutants with defects in chloroplast gene expression show pigment defects and even embryonic lethal phenotypes (Pfalz et al., 2006; Arsova et al., 2010; Chateigner-Boutin et al., 2011; Aryamanesh et al., 2017; Wang et al., 2020). Therefore, chloroplast function maintenance requires the coordinated expression of chloroplast genes, which is important for plant growth and development.

Screening mutants with pigmentation deficient phenotypes is a powerful reverse genetic approach to identify essential genes for chloroplast development. In the past years, several independent research groups have undertaken large-scale genetic screening of the pigmentation deficient mutants and identified a series of nuclear genes essential for chloroplast development (Ajjawi et al., 2010; Myouga et al., 2010). Nevertheless, these findings are still far from a complete understanding of chloroplast development. It is necessary to continue to screen more *Arabidopsis* mutants and identify the corresponding mutated genes, which could provide additional insights into chloroplast biogenesis. In this study, we isolated a novel mutant with an albino lethal phenotype named *RNA processing 8* (*rp8*) in *Arabidopsis* by using T-DNA mutant screening. The *RP8* gene, highly expressed at the seedling stage, encodes a chloroplast-targeted protein. In the *rp8* mutant, the transcription levels of PEP-dependent chloroplast genes are decreased. We subsequently found that the mature transcripts of *rpoA* decreased, which is considered to be the main reason for the deficient transcription of PEP-dependent genes in the *rp8* mutant. These results suggested that RP8 is required for the accumulation of PEP complexes and is involved in chloroplast RNA metabolism. This work would provide a prospect for understanding the role of RP8 in chloroplast RNA metabolism in *Arabidopsis thaliana*.

## Materials and Methods

### Plant Materials and Growth Conditions

*Arabidopsis thaliana* ecotype Columbia [wild type (WT)] was used in this study. The T-DNA insertion line (SALK_080811) was obtained from Arabidopsis Biological Resource Center (ABRC, Ohio State University). The T-DNA insertion site was identified by PCR amplification with the T-DNA left border primer LB3 and the gene-specific primers, Left Primer (LP) and Right Primer (RP). For laboratory work, surface-sterilized seeds were sown on Murashige and Skoog (MS) medium supplemented with 2% sucrose and 0.8% (w/v) agar. Plants were grown at 22°C under 16 h light/8 h dark conditions at 30 μmol photons m^–2^s^–1^.

### Nucleic Acid Isolation, cDNA Synthesis, RT-PCR, qRT-PCR, and Northern Blot Analysis

For genomic DNA isolation, samples were homogenized in extraction buffer [200 mM Tris-HCl, pH 7.5; 25 mM NaCl; 25 mM EDTA;0.5% (w/v) SDS], then the homogenate was extracted through phenol/chloroform. After centrifugation, DNA was precipitated from the supernatant by adding cold isopropyl alcohol. After washing with 70% (v/v) ethanol, the DNA was rehydrated in distilled water. For total RNA extraction, a commercial RNAiso Plus kit (Takara, Otsu, Japan) was used in accordance with the manufacturer’s instructions. Briefly, 2-week-old plants were homogenized in liquid nitrogen, then the homogenate was lysed in the appropriate amount of RNAiso Plus buffer. The mixture was incubated for 5 min at room temperature, then centrifuged at 12,000 rpm for 5 min at 4°C. The supernatant was collected into a new microtube and treated with chloroform. After centrifugation at 12,000 rpm for 15 min at 4°C, an equal volume of isopropanol was added to the supernatant. The pellet was recovered after centrifugation at 15,000 rpm for 10 min at 4°C and dissolved in distilled RNase-free water. Total RNA samples (2–5 μg) were used as templates for the synthesis of the first-strand cDNA with a PrimeScript™ RT Reagent Kit with gDNA Eraser (Takara, Otsu, Japan), according to the manufacturer’s instructions. RT-PCR reactions were performed with specific primers for *RP8*. *Actin2* gene (At3g18780) was used as an internal positive control. Quantitative PCR was performed using the SYBR Green PCR amplification mixture on a StepOnePlus™ Real-Time PCR System (Applied Biosystems, United States). Specific amplification has been confirmed by melting curve analysis. Three biological repeats were performed independently and each sample was operated in triplicate. For Northern blot analysis, equal amounts of total RNA (10 μg) were transferred to positively charged nylon membranes (Roche, Switzerland) after formamide denaturing agarose gel electrophoresis and was further probed with digoxigenin (DIG)-labeled nucleic acid probes (Roche, Switzerland). The probes were synthesized with a PCR DIG synthesis mix (Roche)^1^ with the specific primers listed in Supplementary Table 1. Both hybridization and chemiluminescence detection was carried out, according to the Roche manual.

### Complementation Analysis

As for the genomic complementation analysis, the 2.9 kb full-length genomic fragment of *At4g37920* was amplified using high-fidelity KOD plus polymerase (TOYOBO, Japan)^2^ with the gene-specific primers listed in Supplementary Table 1, and sub-cloned into a binary vector pCAMBIA1301. The construct was introduced into heterozygotes mediated by *Agrobacterium tumefaciens* strain using floral dip transformation as described (Clough and Bent, 1998). Transgenic plants were screened on MS medium with 80 mg L^–1^ hygromycin B (Roche). The genomic backgrounds of these hygromycin-resistant transformants were further analyzed with the specific primers listed in Supplementary Table 1.

### Transmission Electron Microscopy

Leaf segments were fixed with 2.5% glutaraldehyde in phosphate buffer (pH 7.2) for 24 h at 4°C and washed three times with the same buffer. The tissues were postfixed overnight in 1% OsO_4_ at 4°C. The fixed samples were dehydrated through a series of ethanol solutions, infiltrated with a series of epoxy resin in epoxy propane, and embedded in Epon 812 resin. Ultrathin sections were cut with a diamond knife and mounted onto copper grids. Then, the samples were stained with 2% uranyl acetate for 10 min followed by lead citrate for 2 min and observed with a transmission electron microscope (Phillips CM120).

### Fluorescence Microscopy

To construct p35S:RP8-GFP, a 297-bp coding fragment containing the transit peptide was amplified using specific primers and cloned into the *Xho*I and *Spe*I sites of the GFP vector. GFP was transiently expressed in protoplasts of *N. benthamiana* using polyethylene glycol protocol (Lyznik et al., 1991). The GFP fluorescence (green) and chloroplasts autofluorescence (red) of *N. benthamiana* protoplasts were imaged using a confocal laser scanning microscope (Zeiss LSM500). The filter sets used were BP505-545 (excitation 488 nm; emission 505-545 nm) and LP585 (excitation 488 nm; emission 585 nm) to detect GFP and the chlorophyll autofluorescence.

### Protein Extraction and Immunoblot Analysis

Total proteins were extracted from 2-week-old plants, according to the method described (Yu et al., 2011). Different samples were quantified by Protein Assay (Bio-Rad, United States). A total of 30 μg of protein was loaded per lane and separated by 12–15% sodium dodecyl sulfate-polyacrylamide gel electrophoresis (SDS-PAGE). After electrophoresis, proteins were transferred onto a.45 μm PVDF membrane (Millipore, United States) and incubated with antibodies against chloroplast proteins (Agrisera, Sweden).^3^ Signals were identified by an ECL plus Immunoblot detection system (GE) following the manufacturer’s instructions.

### Expression and Purification of the Recombinant RP8 Protein

The coding sequence for the RP8 protein without the N-terminal putative transit peptide was amplified with the KOD plus polymerase (TOYOBO, Japan)^2^ with gene-specific primers listed in Supplementary Table 1 and subcloned into pET51b vector, in frame with a 6 × His tag at C-terminus. The RP8 protein was overexpressed in the *Escherichia coli* BL21 (DE3) strain. When it was grown to OD_600_ = 0.6, 100 mM isopropyl-β-D-thiogalactopyranoside was added. Then, the *E. coli* was cultured at 16°C for 12 h to induce the recombinant protein. All protein purification steps were carried out as described in a study by Sun et al. (2019). The 5 mg purified RP8 protein was sent to Shanghai Orizymes Biotech Company, Shanghai, China to make an antibody of RP8.

## Results

### Isolation and Phenotypic Characterization of the *rp8* Knockout Line

**FIGURE 1:**
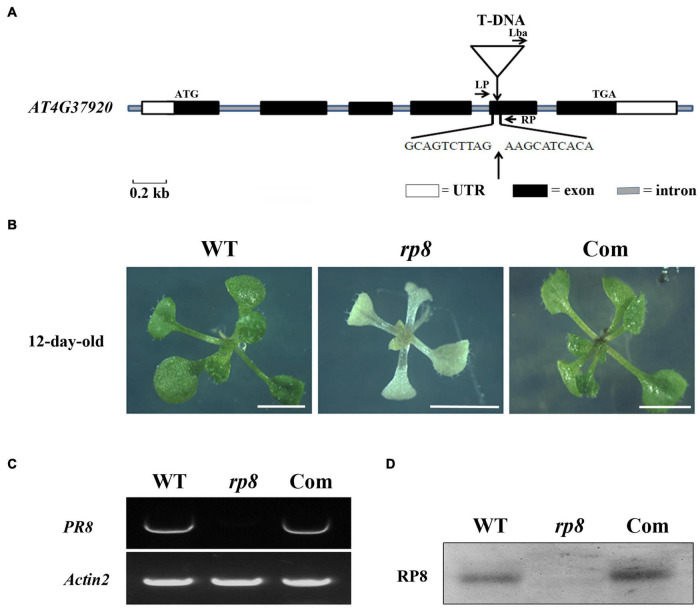
Identification and characterization of the *rp8* mutant. **(A)** Schematic representation of the *RP8* gene and the T-DNA insertion. Black boxes represent exons, lines represent introns, and white boxes represent the start and stop regions. The white triangle shows the T-DNA insertion site in the fifth exon of At4g37920. **(B)** Phenotypes of 12-day-old wild-type (WT), *rp8* mutant, and complementation plant (Com). All plants are grown on MS medium containing 2% sucrose under light intensity at 30 μmol m^– 2^ s^– 1^. Bars = 0.5 cm. **(C)** RT-PCR analysis of the *RP8* gene expression in WT, *rp8* mutant, and complementation plants. *Actin2* was used as an internal control. **(D)** Immunoblot analysis of the RP8 protein in WT, *rp8* mutant, and complementation plants. Each lane was loaded with 30 μg total proteins.

To identify novel nuclear-encoding factors essential for chloroplast development, we screened pigment-deficient mutants of T-DNA insertional lines from the Arabidopsis Biological Resource Center, ABRC. Among the obtained mutant lines, an albino mutant from SALK_080811 with seedling lethal phenotype was characterized and subsequently named as *rp8* (RNA Processing 8). PCR sequencing confirmed that the T-DNA was inserted into the fifth exon of the locus, At4g37920 (Figure 1A) as claimed in the SALK database. Genetic analysis revealed that the progenies from heterozygous segregated approximately at a 3:1 ratio, indicating that the mutant was caused by a single recessive mutation. When cultivated on sucrose-supplemented medium under low light conditions at 30 μmol photons m^–2^s^–1^, homozygous lines still showed an albino phenotype which only can survive for approximately 3 weeks with six to eight leaves (Figure 1B). Examination of young siliques of the heterozygous line also showed that approximately 25% of developing ovules were white (Supplementary Figure 1). RT-PCR results showed that no transcript of the *RP8* gene was detected in the *rp8* mutant, while it was present in the wild type (Figure 1C). Further immunoblot analysis showed that the corresponding size of 43 kDa protein was not detected in the *rp8* mutant, but clearly present in the wild type (Figure 1D).

To further confirm whether the knockout of the At4g37920 locus is responsible for the albino phenotype, we performed the genetic complementation analysis. We cloned a full-length genomic fragment of the At4g37920 gene, then transformed it into the heterozygous lines through *Agrobacterium* transformation. We obtained more than seven independent transgenic complementation lines with *RP8* genotype but showed the wild-type phenotype (Figure 1B). RT-PCR analysis detected the full-length transcript of the *RP8* gene in these complementation lines and a specific 43 kDa protein could also be detected by immunoblot analysis as in wild type (Figures 1C,D). Taken together, these results demonstrated the albino mutant was due to the knockout of the *At4g37920* locus which is essential for photoautotrophic growth and plant viability in *Arabidopsis*.

### *RP8* Encodes a Novel Chloroplast-Localized Unknown Protein in *Arabidopsis*

**FIGURE 2:**
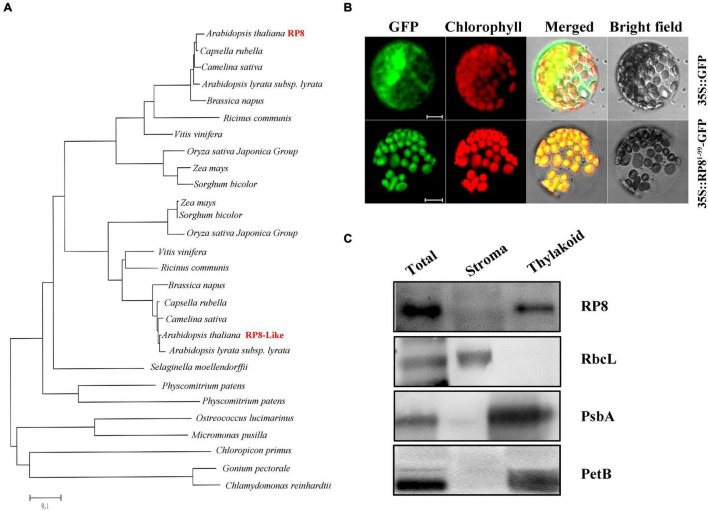
Sequence, phylogenetic analysis, expression pattern, and subcellular localization of the RP8 protein. **(A)** Phylogenetic analysis of the RP8 protein and its homologs. Maximum likelihood analysis of the RP8 protein and its homologs from various organisms was performed. The unrooted phylogenetic tree was constructed by the Neighbor-Joining method (Saitou, 1987) with genetic distance calculated by MEGA 3.1. **(B)** Subcellular localization of RP8. Transit peptide of 99 amino acid residues at the N-terminus of the RP8 protein fused with GFP was transformed into *N. benthamiana* protoplasts by PEG transformation. The red-colored chlorophyll autofluorescence and GFP fluorescence were detected by a laser confocal-scanning microscopy after infiltration. Bars = 5 μm. **(C)** Immunoblot analysis of RP8 in chloroplast subfractions. Stromal and thylakoid proteins fractions were prepared and separated by SDS-PAGE. Immunoblot analysis using antibodies against RbcL, RP8, PsbA, and PetB was performed. Each lane was loaded with 30 μg proteins.

The genome locus *At4g37920* encodes a 427-aa-long protein with a predicted molecular mass of 48.7 kDa. It was annotated as an endoribonuclease E-like protein (RNase E-like) in the TAIR database.^4^ Nevertheless, alignment analysis revealed no significant similarity between the RP8 and RNase E proteins, and conserved domain analysis also showed no known information in the currently available database (see text footnote 5). This analysis indicated that *RP8* encoded an unknown protein, and it does not belong to the endoribonuclease E-like family. Sequence searching of the PPDB database identified that one homologous of RP8, RP8-like protein (AT1G36320), is probably located in the chloroplast of *Arabidopsis*. They share 41% identity and 67% similarity at the amino acid level^5^ (Supplementary Figure 2). No obvious functional domain or motif was predicted in either RP8 or the RP8-like protein by any available bioinformatic tools. The BLASTP searching database in the NCBI website revealed that RP8 and the RP8-like protein are present in both dicotyledons and monocotyledons, such as *Camelin sative*, *Brassica napus*, *Capsella rubella*, *Ricinus communis and Oryza*, *Sorghum bicolor*, *Zea mays*, and *Physcomitrium patens*. But only RP8-like homologous proteins are found in the species including S*elaginella moellendorffii*, moss, and a green alga. No homologous protein is detected in bacteria (Figure 2A). These data suggested that RP8 and its homologous proteins are widely present in green plants, which probably originated from green alga.

Both the TargetP^6^ (Emanuelsson et al., 2000) and Predator software predicted that the RP8 protein contains a putative chloroplast trans-peptide at the N-terminal region. To further confirm the subcellular localization of RP8, we constructed the coding sequence of the N-terminal 99-amino acid fused to a green fluorescent protein (GFP) under the control of the cauliflower mosaic virus 35S promoter. The plasmid containing the chimeric gene was transformed into the protoplasts of *N. benthamiana* through a polyethylene glycol (PEG)-mediated transformation method. Transient expression of the fusion protein was then examined by a confocal laser-scanning microscope. Our results showed that the green fluorescence of the chimeric protein was co-localized with chlorophyll autofluorescence (Figure 2B). By contrast, the fluorescence from free GFP protein was present ubiquitously in the cytoplasm. These data suggested that the N-terminal region functions as a trans-peptide that is able to target the RP8 protein to the chloroplast exclusively. Thus, the RP8 protein is localized in the chloroplast. To further demonstrate its location in the chloroplast, the stroma and thylakoid fractions were separated and immunoblot analysis using antibodies against corresponding marker proteins of stroma (RbcL) and thylakoid (PsbA and PetB) were performed. These results indicated that RP8 is associated with the thylakoid membranes (Figure 2C). Taken together, we concluded that RP8 is a chloroplast-localized protein associated with the thylakoid membranes in *Arabidopsis*.

### Expression of the *RP8* Gene in *Arabidopsis*

**FIGURE 3:**
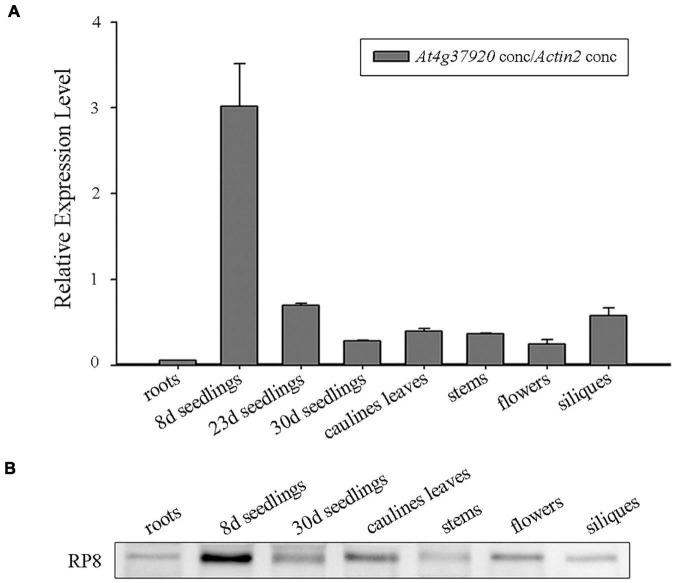
Expression analysis of the *RP8* gene. **(A)** Relative expression of the *RP8* gene in different tissues of *Arabidopsis*. Total RNA was extracted from various tissues of the wild type and RT-qPCR was carried out. All values are means of three biological replicates with SD. **(B)** Detection of the RP8 protein in extracts of different tissues in *Arabidopsis*. Each lane was loaded with 15 μg proteins.

We examined the expression of the *RP8* gene using the publicly available microarray data and Genevestigator v3 (Zimmermann et al., 2004). The developmental expression analysis revealed that the *RP8* gene is highly expressed in seedlings, leaves, and flowers (Supplementary Figure 3). The anatomical expression analysis showed a higher expression of the *RP8* gene in juvenile leaves compared with other tissues, suggesting that *RP8* plays an important role during the early stage of chloroplast development (Supplementary Figure 3). To investigate the expression pattern of the *RP8* gene, total RNA was extracted from various tissues of the wild type, quantitative reverse transcription RT-qPCR analysis was performed. Our results showed that the *RP8* gene was expressed in leaves, stems, flowers, siliques, and especially high in 8-day-old seedlings. In contrast, the *RP8* transcript in roots was barely detected. This result suggests that the *RP8* gene is widely expressed in photosynthetic tissues (Figure 3A). We also used an antibody against RP8 to confirm its accumulation in different plant tissues. As Figure 3B showed, the RP8 protein accumulated highly in the 8-day-old seedlings, which is consistent with the transcript levels. Unexpectedly, the RP8 protein can still accumulate in roots, although transcripts of RP8 were barely detected in roots. Taken together, our data showed that the *RP8* gene is strongly expressed in photosynthetic tissues at both transcription and protein levels.

### Defects in Chloroplast Development in the *rp8* Mutant

**FIGURE 4:**
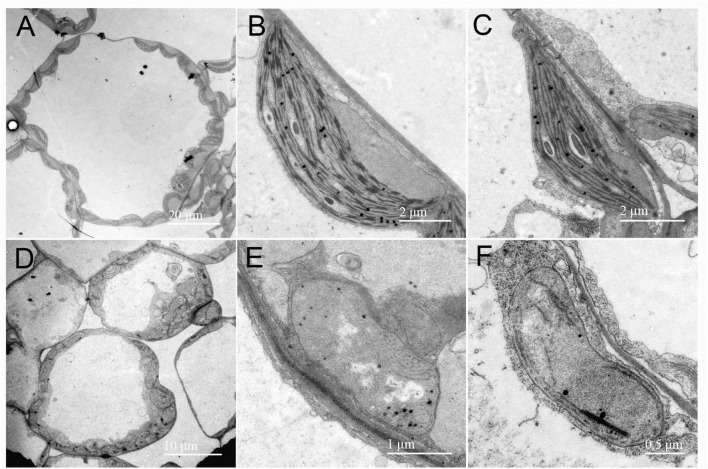
Chloroplast ultrastructure observation in the wild type and the *rp8* mutant. Transmission electron micrographs of chloroplasts in leaves from the 12-day-old wild type **(A–C)** and the *rp8* mutant **(D–F)** grown under low light intensity at 30 μmol photons m^– 2^ s^– 1^. **(A,D)** Are the overview of mesophyll cell chloroplasts. **(B,E)** Are chloroplasts of cotyledons, **(C,F)** Are chloroplasts of true leaves. Scale bars are indicated.

**FIGURE 5:**
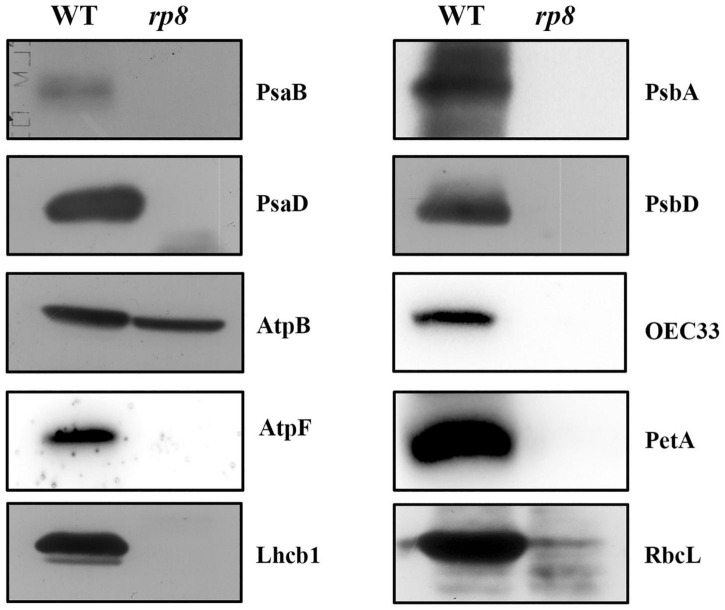
Accumulation of photosynthetic proteins in the wild type and the *rp8* mutant. Immunoblot analysis of photosynthetic proteins PsaB, PsaD, AtpB, AtpF, Lhcb1, PsbA, PsbD, OEC33, PetA, and RbcL from the wild type (WT) and the *rp8* mutant using the corresponding antibodies, respectively. Each lane was loaded with 30 μg total proteins.

To examine the development status of the chloroplast in the *rp8* mutant, we observed the chloroplast from 2-week-old seedlings by transmission electron microscopy (TEM). In the wild type, the chloroplast contains a well-organized stroma and stacked grana thylakoids in both cotyledons and true leaves (Figures 4A–C). In contrast, the thylakoids were nearly absent in the cotyledons of the *rp8* mutant, and only a few thylakoid lamellae can be observed from its true leaves (Figures 4D–F). These observations indicated that chloroplast development in the *rp8* mutant was seriously arrested at the early stage and that RP8 is essential for thylakoid formation and chloroplast development in *Arabidopsis*. We also investigated the accumulation of photosynthetic-related proteins in the *rp8* mutant. These proteins, including three components of PSII complex (PsbA, PsbD, and OEC33), two components of PSI (PsaB and PsaD), one component of cy*b6/f* complex (PetA), two ATP synthase subunits (AtpB and AtpF), one subunit of light-harvest complex and chlorophyll a/b-binding protein (Lhcb1), and the large subunit of rubisco (RbcL), were checked using their corresponding antibodies. Our results showed that the amounts of AtpB and RbcL were reduced in the *rp8* mutant (Figure 5 and Supplementary Figure 5). PsbA, PsbD, OEC33, PsaB, PsaD, and Lhcb1 are barely detected in the *rp8* mutant, although these proteins can be detected in the wild type. These results indicate that components of photosynthetic complexes were seriously reduced in the *rp8* mutant. Altogether, these results prove that the accumulation of photosynthetic proteins and the development of chloroplasts were seriously blocked in the *rp8* mutant.

### Expression of Chloroplast-Associated Genes in the *rp8* Mutant

**FIGURE 6:**
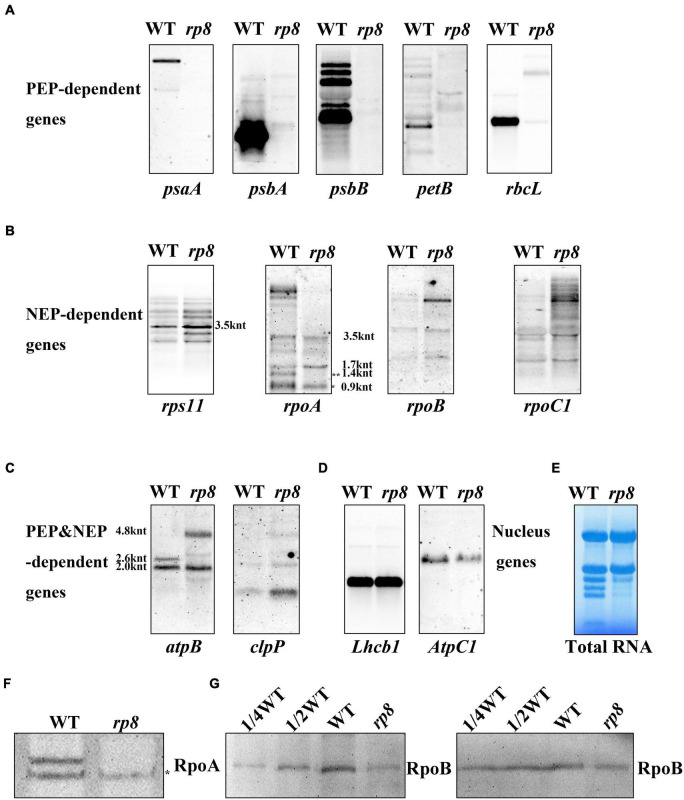
Northern blot analysis of chloroplast-associated genes in the wild type (WT) and the *rp8* mutant. **(A)** Steady-state levels of PEP-dependent transcripts, *psaA*, *psbA*, *psbB*, *petB*, and *rbcL*. **(B)** Steady-state levels of NEP-dependent transcripts (*rps11*, *rpoA*, *rpoB*, and *rpoC1*). * Indicates the mature transcripts of *rpoA* (990 nt), ** indicates the 1.4 knt transcript as described in Zhang et al. (2015). The sizes of the transcripts were indicated according to Wu and Zhang (2010). **(C)** Steady-state levels of chloroplast transcripts that are both PEP- and NEP-dependent (*atpB* and *clpP*). As for the *atpB* transcripts, 4.8, 2.6, and 2.0 knt transcripts are indicated. **(D)** Steady-state levels of two nucleus genes (*Lhcb1* and *AtpC1*). **(E)** Methylene blue staining of total RNA for loading control. About 10 μg total RNA was transferred to a nylon membrane after electrophoresis, then probed with DIG-labeled probes. **(F)** Immunoblot analysis of RpoA with the antibody described in Zhang et al. (2018) (*represents the specific band for the RpoA protein). **(G)** Immunoblot analysis of RpoB protein levels in the wild type (WT) and the *rp8* mutant. Total proteins from the WT samples were loaded with three different concentrations (7.5, 15, and 30 μg), and total proteins from the mutant samples were loaded with 30 μg, respectively. *Arabidopsis* mutant *ptac10* with a decreased RpoB protein level was used as a control (Chang et al., 2017; Yu et al., 2018).

Because numerous nucleus-encoded genes regulate chloroplast gene expression (Zhou et al., 2009; Chateigner-Boutin et al., 2011; Gao et al., 2011; Yu et al., 2013; Huang et al., 2018; Wang et al., 2020), we - want to check the putative role of RP8 in chloroplast RNA metabolism by investigating chloroplast transcript profiles in the *rp8* mutant by Northern blot. The *psaA*, *psbA*, *psbB*, *petB*, and *rbcL* genes were chosen because they are PEP-dependent (class I). The *rps11*, *rpoA*, *rpoB*, and *rpoC2* were chosen because they are NEP-dependent (class III). The *atpB* and *clpP* were chosen because they are both PEP and NEP-dependent (class II) (Hajdukiewicz et al., 1997). As for those PEP-dependent chloroplast transcripts, their accumulations were seriously reduced in the *rp8* mutant compared with those of the wild type (Figure 6A). In contrast, the accumulations of NEP-dependent transcripts (*rps11*, *rpoB*, *rpoC2*) were increased in the *rp8* mutant (Figure 6B). As for the *atpB* operon, 2.6- and 2.0-kb transcripts are transcribed by PEP (σ dependent) and NEP, respectively (Schweer et al., 2006). In the *rp8* mutant, the 2.6-kb transcripts observed in the wild-type plant are not transcribed, whereas a novel 4.8-kb transcript transcribed by NEP was detected in the *rp8* mutant (Figure 6C). We also checked the transcripts of two nucleus genes *Lhcb1* and *AtpC1*, and no significant alteration was detected between the *rp8* mutant and the wild type (Figure 6D). This expression pattern is similar to other PEP-deficient mutants that have been reported earlier (Pfalz et al., 2006; Gao et al., 2011; Yu et al., 2013). Thus, our data showed that the null mutation of *RP8* affected the proper expression of chloroplast genes, resulting in reduced amounts of PEP-dependent chloroplast transcripts.

Noticeably, the *rpoA* gene encoding the alpha subunit of the PEP complex was transcribed by NEP. Here, we found that the number of mature transcripts of *rpoA* (about 990 nt) was clearly decreased in the *rp8* mutant. In contrast, no obvious difference in the processing of the *rps11* transcript located in the *rpoA* polycistron was observed between the wild type and the *rp8* mutant. Since the accumulation of mature transcripts of *rpoA* was decreased in the *rp8* mutant, we further checked whether the level of the RpoA protein was affected. Immunoblot analysis showed that the protein level of RpoA in the mutant was dramatically reduced by 50% (Figure 6F). This result suggests that the defective *rpoA* polycistronic processing affects the accumulation of the RpoA protein. Similarly, we investigated the accumulation of another core protein of the PEP complex, RpoB, in the *rp8* mutant. Our data showed that the RpoB protein level was also dramatically reduced by 25% in the *rp8* mutant (Figure 6G). Taken together, our data suggested that the RP8 deletion affects the processing of *rpoA* transcripts, which resulted in the defective accumulation of the PEP core complex and impaired expression of PEP-dependent chloroplast genes at the early growth stage.

### Ribosomal RNAs and Proteins Were Substantially Reduced in the Chloroplast of the *rp8* Mutant

**FIGURE 7:**
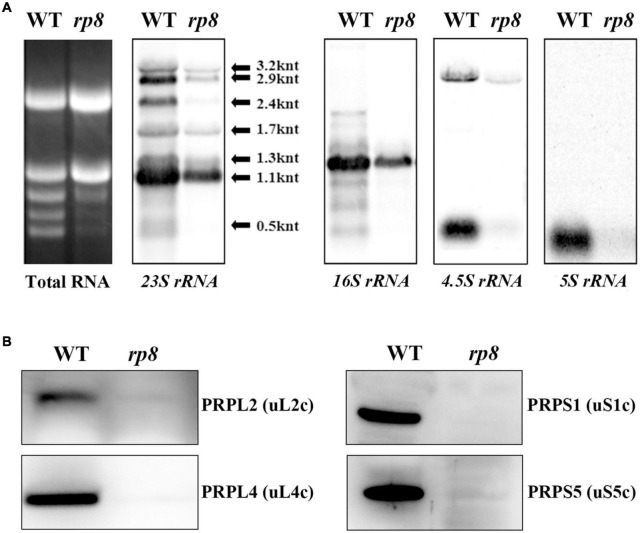
Analysis of chloroplast ribosomes in the wild type (WT) and the rp8 mutant. **(A)** Total RNA in the WT and the *rp8* mutant and Northern blot analysis of chloroplast-encoded rRNAs. **(B)** Immunoblot analysis of chloroplast ribosomal proteins PRPS1 (uS1c), PRPS5 (uS5c), PRPL2 (uL2c), and PRPL4 (uL4c) in the WT and the *rp8* mutant. Each lane was loaded with 30 μg total proteins.

Ribonucleic acid processing 8 (RP8) is associated with thylakoids, as translationally active ribosomes are (Zoschke and Barkan, 2015). In the *rp8* mutant, accumulation of both alpha- and betta-PEP subunits (RpoA and RpoB) are affected (Figures 6F,G), and photosynthetic membrane proteins are barely detected (Figure 5). We further checked the possibility of RP8 interference with translation. First, we checked the accumulation of chloroplast rRNAs in the *rp8* mutant compared with that of the wild type. Northern blot analysis showed that the abundance of the chloroplast rRNA, including *23S*, *16S*, *4.5S*, and *5S* rRNA, was severely decreased in the *rp8* mutant (Figure 7A). Second, we checked the amount of chloroplast ribosome proteins in the *rp8* mutant. Our immunoblot analysis showed that the amounts of chloroplast ribosome proteins, including PRPS1 (uS1c), PRPS5(uS5c), PRPL2 (uL2c), and PRPL4 (uL4c), were substantially reduced in the *rp8* mutant (Figure 7B), compared with those in the wild type. These data showed that knockout of *RP8* seriously affected chloroplast translational machinery.

## Discussion

In this study, we characterized a pigmentation-deficient mutant *rp8* in detail. Our data demonstrated that the chloroplast-localized RP8 protein is required for the maturation of the polycistronic *rpoA* transcript, which is essential for PEP function and chloroplast development. Our work would enrich the knowledge in chloroplast gene expression and chloroplast development.

In higher plants, chloroplasts come from proplastids which are small undifferentiated plastids lacking pigments or internal membrane structures. This process is often accompanied by the highly coordinated expression of plastid- and nuclear-encoded genes (Shiina et al., 2005), which is necessary to maintain chloroplast function. Since most chloroplast proteins are nuclear-encoded, many nuclear genes mutations result in a defect in chloroplast development and albino lethal phenotype (Pfalz et al., 2006; Arsova et al., 2010; Gao et al., 2011; Yu et al., 2013; Wang et al., 2020). Although RP8 encoded by the At4g37920 gene in *Arabidopsis* is annotated as an RNase E-like protein, no further evidence supported this annotation at present. The albino lethal phenotype of the *rp8* mutant may result from the arrested chloroplast development since they have poorly developed abnormal chloroplast with a few thylakoids lamella (Figure 4). Accordingly, the accumulation of thylakoid proteins in the *rp8* mutant was substantially reduced (Figure 5). Genetic complementation analysis confirmed that the loss of function of the *RP8* gene is required for the mutant phenotype. Consistent with the transcription level of the *RP8* gene, the RP8 protein is highly expressed at the seedling stage, suggesting an essential role for the RP8 protein in chloroplast development (Figure 3). Although the transcript level of RP8 in roots was low, the RP8 protein can still accumulate in roots, and its abundance is no different from that in stems, flowers, and siliques. There may exist a distinct regulator at the expression level of the *RP8* gene in these tissues.

Phylogenetic analysis showed that the RP8 protein probably originated from the green alga, not from cyanobacteria (Figure 2A). Both RP8 and the RP8-like protein are present in higher plants but only RP8-like proteins exist in a green alga, indicating gene duplication probably happened during the evolution of organisms from ocean to land. Although the RP8 protein shares high identity with the RP8-like protein (Supplementary Figure 2), the pleiotropic phenotype of the *rp8* mutant strongly suggested its distinct function in chloroplasts of higher plants, which also highlighted the importance of the RP8 protein family during chloroplast development. In *Arabidopsis* chloroplasts, the existence of RP8 and its paralogous proteins appear not to be occasional. For example, MRL7 and MRL7-L are two homologous chloroplast proteins that play essential but non-redundant roles (Qiao et al., 2011). Both MRL7 and MRL7-L are present in embryophytes, but MRL7-L is absent in lower plants (Qiao et al., 2011; Yu et al., 2011, 2014b). Although both MRL7 and MRL7-L are involved in regulating chloroplast gene expression, knockout of either of them results in an albino phenotype (Qiao et al., 2011), indicating the functional diversification of MRL7 and MRL7-L in chloroplasts.

Chloroplast gene expression is required for the development of chloroplast in higher plants. Loss-of-function mutation genes encoding subunits of chloroplast RNA polymerase and their regulatory proteins always impaired chloroplast gene expression and further resulted in arrested chloroplast development (Pfalz et al., 2006; Gao et al., 2011; Yu et al., 2013). In this work, we investigated the expression profiles of chloroplast genes in the *rp8* mutant. Northern blot results showed that the *rp8* mutant displayed seriously reduced levels of chloroplast-encoded photosynthetic genes transcribed by PEP, but the transcript levels of NEP-dependent genes, *rpoB*, *rpoC1* were increased (Figures 6A–C). The increased levels of NEP-dependent chloroplast gene expression in the mutant might be due to a feedback regulatory mediated by tRNA*^Glu^* (Hanaoka et al., 2005). This expressional pattern of chloroplast transcripts in the *rp8* mutant resembles those of *paps* mutants and Δ*rpo* mutants (Pfalz et al., 2006; Ajjawi et al., 2010; Myouga et al., 2010; Gao et al., 2011; Yu et al., 2013; Chang et al., 2017; Liebers et al., 2018). These results suggest that RP8 probably plays an important regulatory role in chloroplast gene expression. Nevertheless, no evidence showed that the RP8 protein is a component of the PEP complex, like PAPs proteins (Pfalz et al., 2006; Steiner et al., 2011). It was noticeable that the mature transcript of *rpoA* (990 nt) located in the *L23-L2-S19-L22-S3-L16-L14-S8-L36-S11-rpoA* polycistron was decreased in the *rp8* mutant. But the transcripts of *rps11* in the same polycistron upstream of *rpoA* were not affected (Figure 6B), indicating that RP8 may specifically be involved in the processing of *rpoA* transcripts. In addition, we checked the effects of the *rp8* mutation on the intron splicing of chloroplast genes. There are no obvious abnormal chloroplast pre-mRNA splicing events found in the *rp8* mutant (Supplementary Figure 4), suggesting that RP8 may be specifically involved in the processing of the *rpoA* polycistron in chloroplasts. Like the *opt70* mutant in *Arabidopsis*, interrupting the processing of the *rpoC1* transcript which encodes the β′ core subunit of the PEP complex impaired the PEP activity and led to the defective chloroplast development (Chateigner-Boutin et al., 2011). Our data suggested that reduced *rpoA* transcripts affected the accumulation of the RpoA protein in the *rp8* mutant, which may result in decreased accumulation of the PEP complex and subsequently affect the expression of PEP-dependent chloroplast genes (Figure 6).

Interestingly, we found some other nuclear-encoded proteins associated with the *rpoA* transcript in *Arabidopsis*, which leads to a lower accumulation level of the RpoA protein and reduced transcription of PEP-dependent genes. For example, mTERF6 is directly associated with a 3′-end sequence of the *rpoA* polycistron and is involved in the transcription termination of the *rpoA* polycistron (Zhang et al., 2018). The PDM1 protein is involved in the processing of *rpoA* transcripts by associating with the intergenic sequence of *S11-rpoA*, which is important for post-transcriptional regulation in chloroplasts (Wu and Zhang, 2010; Yin et al., 2012; Pyo et al., 2013; Zhang et al., 2015). It further suggested that PDM1 is a hub protein; it can recruit various functional proteins which are responsible for different post-transcriptional processing (Yin et al., 2012; Zhang et al., 2015). So it is reasonable for us to speculate that the RP8 protein may be recruited by some *rpoA*-associated proteins to assist related processes during *rpoA* transcript processing. Of course, this assumption still needs to be further investigated in the future. Additionally, RP8 is associated with thylakoids (Figure 2C), as translationally active ribosomes (Zoschke and Barkan, 2015). We found that both chloroplast rRNAs and chloroplast ribosomal proteins were significantly decreased in the *rp8* mutant (Figure 7), which suggested that the function of RP8 might be related to chloroplast translation. We cannot exclude the possibility that the reduction of chloroplast rRNAs and chloroplast ribosomal proteins is due to the pleiotropic effects of the *RP8* mutation.

## Data Availability Statement

The datasets presented in this study can be found in online repositories. The names of the repository/repositories and accession number(s) can be found in the article/Supplementary Material.

## Author Contributions

MK, QY, and HM designed the experiments and wrote the manuscript. MK, YW, ZW, and WQ performed the experiments. YXL, XC, and YYL analyzed the data. PS and ZY analyzed the data and revised the manuscript. All authors contributed to the article and approved the submitted version.

## Conflict of Interest

The authors declare that the research was conducted in the absence of any commercial or financial relationships that could be construed as a potential conflict of interest.

## Publisher’s Note

All claims expressed in this article are solely those of the authors and do not necessarily represent those of their affiliated organizations, or those of the publisher, the editors and the reviewers. Any product that may be evaluated in this article, or claim that may be made by its manufacturer, is not guaranteed or endorsed by the publisher.

## Footnotes

1
http://www.roche.com

2
http://www.toyobo-global.com/

3
http://www.agrisera.com

4
https://www.arabidopsis.org/

5
http://ppdb.tc.cornell.edu/dbsearch/gene.aspx?acc=AT1G36320

6
http://www.cbs.dtu.dk/services/TargetP/

## Supplementary Material

Supplementary Figure 1 The seedling-lethal phenotype of the *rp8* mutant.

Supplementary Figure 2 Protein alignment analysis between RP8 and RP8-like Protein (AT1G36320).

Supplementary Figure 3 Different stages of development and tissue expression of the *RP8* gene, according to publicly available Affymetrix GeneChip microarray data.

Supplementary Figure 4 RT-PCR analysis of intron splicing of chloroplast genes in the wild type and the *rp8* mutant.

Supplementary Figure 5 Coomassie brilliant blue staining for the wild type and the *rp8* mutant.
