# Supplementary material for: A Novel Chloroplast Protein RNA Processing 8 Is Required for the Expression of Chloroplast Genes and Chloroplast Development in Arabidopsis thaliana
Source: Front Plant Sci. 2021 Dec 9;12:700975. doi: 10.3389/fpls.2021.700975 (PMC8695849; doi:10.3389/fpls.2021.700975)
Supplement: Supplementary file 1 [file Table_1.pdf]

Supplemental Table S1. Primers used in this study.

Names	Primer sequence 5'→3' (forward / reverse)
T-DNA left border primer LB3	ATTTTGCCGATTTCGGAAC
LP	GGACCCTACATGGGCTTATTG
RP	CAGAAACTCTTGATGGCTTGG
LPP (RP+LPP to check the hygromycin-resistant complementation plants）	TATTGATCTCATAGCCAGGCC
complementation F	GGTACCTTTCTCTATTACATCCTTCTATGG
complementation R	CTGCAGTCAGTTCAAAAAATCTTCAATAGT
RT-PCR RP8-F	AGGAGCTTGATTCCTCATTGA
RT-PCR RP8-R	TTAGGGTCCTTAGCTTCGTG
qRT-PCR RP8-F	TCAAACCCATCATCTCCCTT
qRT-PCR RP8-R	GAGCCATAGTGTAACCTTCAGC
RT-PCR ACTIN2 F	TCTTCTTCCGCTCTTTCTTTCC
RT-PCR ACTIN2 R	TCTTACAATTTCCCGCTCTGC
qRT-PCR ACTIN2 F	TTGAATCTCCGGCGACTTGA
qRT-PCR ACTIN2 R	CCAACATATGCATCCTTCTG
YFPF	CTCGAGATGGCGAATTTACTGGAAACA
YFPR	ACTAGTAGGTTTCTCATTCAAGAAGAGAT
RP8 PROTEIN F	GGTACCTGCGG AAGTAAAAAGCTCTGTT
RP8 PROTEIN R	AAGCTTTCAGTTCAAAAAATCTTCAATA
psaA F for northern blot	GCGAGCACCAGTTTGACTTG
psaA R for northern blot	CCTACTGCAATAATTCTTGC
psbA F for northern blot	ACAACATTGTAGCTGCTCAC
psbA R for northern blot	CTAACACTAACGAATTATCC
psbB F for northern blot	ATGGGTTTGCCTTGGTATCG
psbB R for northern blot	ACAATTCCGTCTCCGTCTAC
petB F for northern blot	GGTTCGAAGAACGTCTTGAG
petB R for northern blot	AGGGACCAGAAATACCTTGC
atpB F for northern blot	ATGAGAACAAATCCTACTAC

atpB R for northern blot	GCCTTCGCAGTAGCTTCATC
rbcL F for northern blot	TCCAACGCATAAATGGTTGG
rbcL R for northern blot	CTAAAGCAAGTGTTGGGTTC
ClpP F for northern blot	ATGATCCATCAACCCGCTAG
ClpP R for northern blot	TATTGAACCGCTACAAGATC
rps11 F for northern blot	AAACCTATATTAAGAATTGG
rps11 R for northern blot	TATACACGTCTTTTTTTAGG
rpoA F for northern blot	CGGACACTACAGTGGAAGTG
rpoA R for northern blot	TGAATACAGCATCGATAGG
rpoB F for northern blot	ATGCCTTATTTGCAAGACGG
rpoB R for northern blot	GATCGTAGTTCTCGAACAAG
rpoC2 F for northern blot	AATAATTCGCCAGATTCGGG
rpoC2 R for northern blot	AGTCACAAAATTCCCTGTGG
Lhcb1 F for northern blot	ATGGCCGCCTCAACAATGGCTC
Lhcb1 R for northern blot	TCACTTTCCGGGAACAAAGTTG
atpC1 F for northern blot	ATGGCTTGCTCTAATCTAACAA
atpC1 R for northern blot	GGAACATCGACATCATCGGTTT
23S rRNA F for northern blot	GGAGTTGAAAATAAGCGTAGATC
23S rRNA R for northern blot	TACTACTTAGATGCTTTCAGCAG
16S rRNA F for northern blot	TCTCATGGAGAGTTCGATCCTG
16S rRNA R for northern blot	TACGGCTACCTTGTTACGACTTC
4.5S rRNA F for northern blot	TCACGGCGAGACGAGCCGTTTAT
4.5S rRNA R for northern blot	GTTCAAGTCTACCGGTCTGTTAG
5S rRNA F for northern blot	TATTCTGGTGTCCTAGGCGTAGA
5S rRNA R for northern blot	ATCCTGGCGTCGAGCTATTTTTC
