# Supplementary figures and images for: A Novel Chloroplast Protein RNA Processing 8 Is Required for the Expression of Chloroplast Genes and Chloroplast Development in Arabidopsis thaliana
Source: Front Plant Sci. 2021 Dec 9;12:700975. doi: 10.3389/fpls.2021.700975 (PMC8695849; doi:10.3389/fpls.2021.700975)

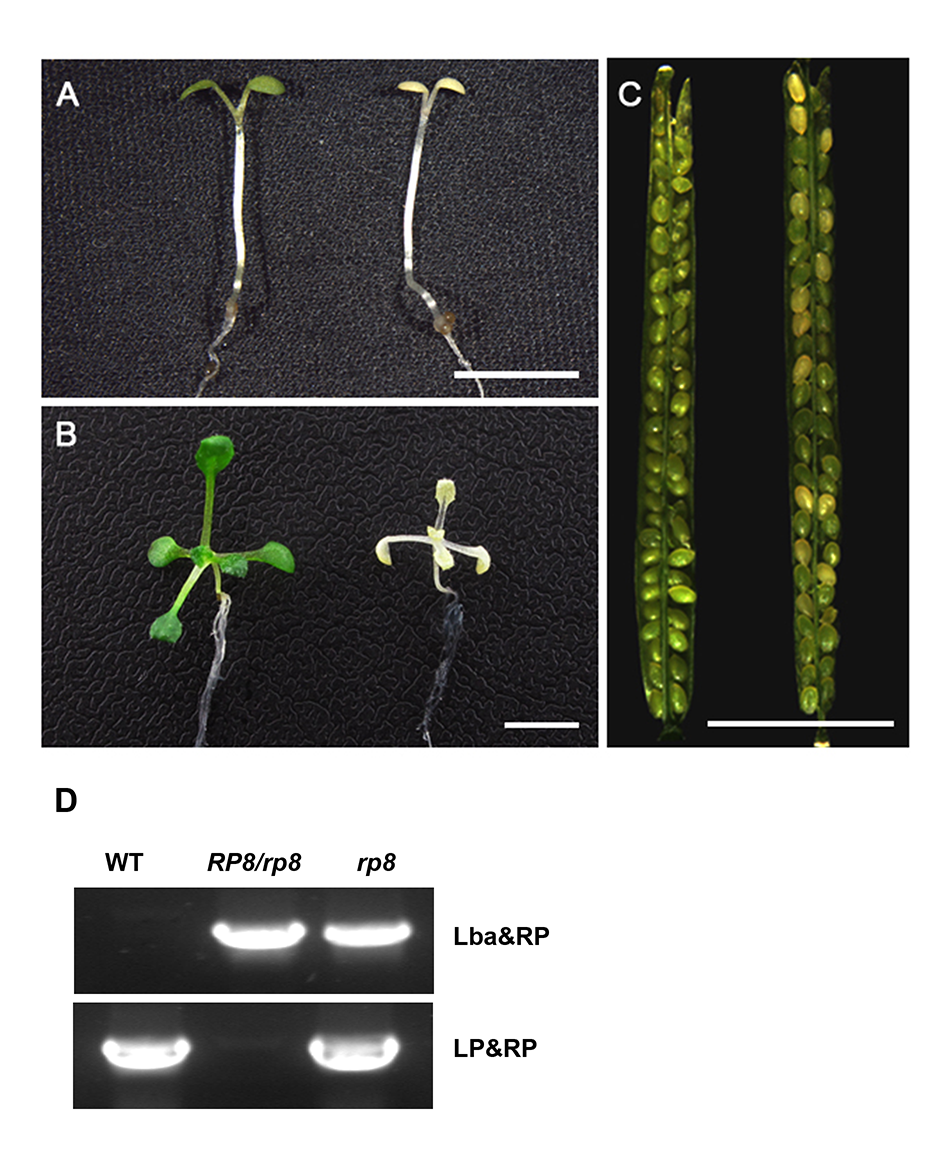

Supplement: Supplementary Figure 1 — The seedling-lethal phenotype of the rp8 mutant. [file Image_1.TIF]

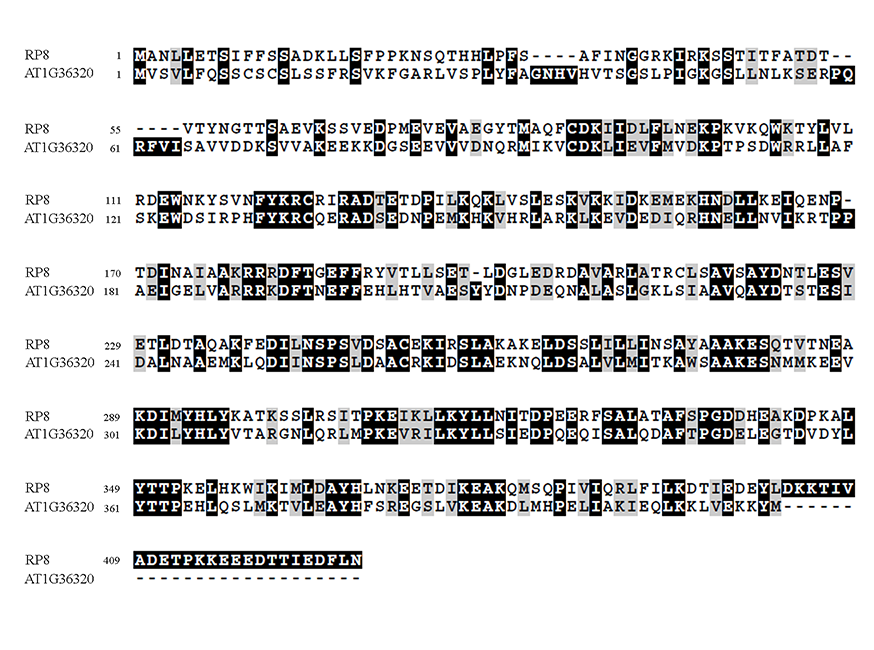

Supplement: Supplementary Figure 2 — Protein alignment analysis between RP8 and RP8-like Protein (AT1G36320). [file Image_2.TIF]

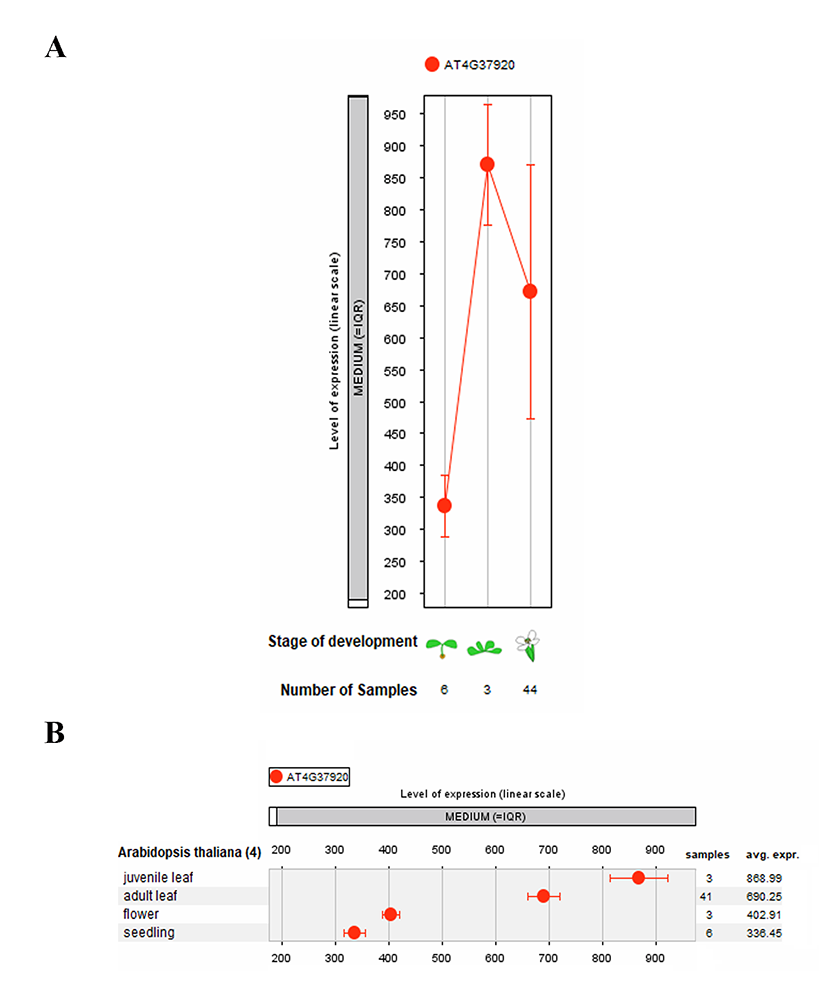

Supplement: Supplementary Figure 3 — Different stages of development and tissue expression of the RP8 gene, according to publicly available Affymetrix GeneChip microarray data. [file Image_3.TIF]

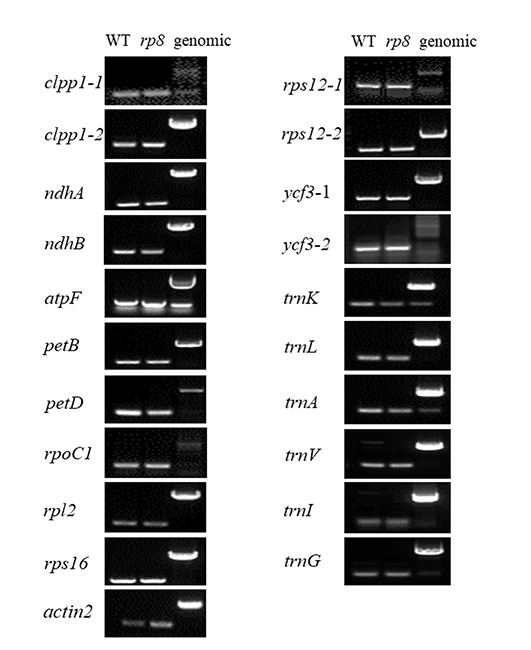

Supplement: Supplementary Figure 4 — RT-PCR analysis of intron splicing of chloroplast genes in the wild type and the rp8 mutant. [file Image_4.TIF]

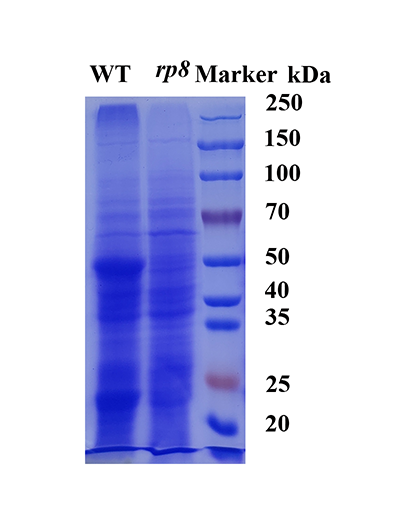

Supplement: Supplementary Figure 5 — Coomassie brilliant blue staining for the wild type and the rp8 mutant. [file Image_5.TIF]
